# Unveiling signaling pathways inducing MHC class II expression in neutrophils

**DOI:** 10.3389/fimmu.2024.1444558

**Published:** 2024-09-30

**Authors:** Pascal Forrer, Darya Palianina, Claudia Stühler, Matthias Kreuzaler, Julien Roux, Jiagui Li, Christoph Schmutz, David Burckhardt, Fabian Franzeck, Daniela Finke, Alexander Schmidt, Dirk Bumann, Nina Khanna

**Affiliations:** ^1^ Department of Biomedicine, University and University Hospital of Basel, Basel, Switzerland; ^2^ Bioinformatics Core Facility, Department Biomedicine, University and University Hospital of Basel, Basel, Switzerland; ^3^ Swiss Institute of Bioinformatics, Department Biomedicine, Basel, Switzerland; ^4^ Focal Area Infection Biology, Biozentrum, University of Basel, Basel, Switzerland; ^5^ Biozentrum, University of Basel, Basel, Switzerland; ^6^ Department of Biomedicine, University and University Children’s Hospital of Basel, Basel, Switzerland; ^7^ Proteomics Core Facility, Biozentrum, University of Basel, Basel, Switzerland; ^8^ Division of Infectious Diseases and Hospital Epidemiology, University Hospital of Basel, Basel, Switzerland

**Keywords:** innate immunity, antigen-presenting cells, APC-like neutrophils, gram-negative bacteremia, sepsis, JAK-STAT signaling, MHC class II, GM-CSF

## Abstract

**Introduction:**

Gram-negative bacillary bacteremia poses a significant threat, ranking among the most severe infectious diseases capable of triggering life-threatening sepsis. Despite the unambiguous involvement of neutrophils in this potentially fatal disease, there are limited data about the molecular signaling mechanisms, phenotype, and function of human neutrophils during the early phase of gram-negative bacillary bacteremia.

**Methods:**

By using an unbiased proteomics and flow cytometry approach, we identified an antigen-presenting cell (APC)-like phenotype in human peripheral blood neutrophils (PMN) with MHC class II molecule expression in the early phase of bacteremia. Using an in-vitro model of GM-CSF-mediated induction of APC-like phenotype in PMN, we investigated downstream signaling pathways leading to MHC class II expression.

**Results:**

GM-CSF stimulation of neutrophils leads to the activation of three major signaling pathways, the JAK-STAT, the mitogen-activated protein kinase (MAPK), and the phosphoinositide 3-kinase (PI3K)-Akt-mTOR pathways, while MHC class II induction is mediated by a MAPK-p38-MSK1-CREB1 signaling cascade and the MHC class II transactivator CIITA in a strictly JAK1/2 kinase-dependent manner.

**Discussion:**

This study provides new insights into the signaling pathways that induce MHC class II expression in neutrophils, highlighting the potential for therapeutic targeting of JAK1/2 signaling in the treatment of gram-negative bacteremia and sepsis. Understanding these mechanisms may open up novel approaches for managing inflammatory responses during sepsis.

## Introduction

Gram-negative (GN) bacteremia is one of the most serious infectious diseases (1), which may result in sepsis defined as a life-threatening organ dysfunction caused by a dysregulated host response to infection (2). GN bacteremia mortality rates vary between 12% and 38% (3–5), depending on patients’ age and underlying diseases (6), on whether the patient receives timely and appropriate antibiotic therapy (4), and on growing bacterial resistance to antibiotics (7, 8). The World Health Organization identified sepsis as a global health priority; however, attempts to develop new treatments failed in large clinical trials (9). Gram-negative bacillary sepsis thus presents a major unmet medical need.

To identify and develop novel control strategies, a detailed understanding of the host-related factors of Gram-negative bacillary bacteremia and sepsis is essential. The early phase involves excessive inflammation (10). Neutrophils as first-line defense against invading pathogens play an explicit role during the early phase to recognize, phagocytose, and kill pathogens. Pathogen elimination depends on neutrophil recruitment to the site of infection (11). In addition to these beneficial responses, neutrophils can contribute to the development of multiple-organ failure in sepsis (12) most likely by aberrant regulation of NETosis (13). Moreover, neutrophils are able to interact with other cell types, e.g., lymphocytes (14), and shape many facets of the immune response (15). Neutrophils can traffic antigens to lymph nodes, where they express both classes of major histocompatibility proteins (MHC class I and class II) (16–19) and co-stimulatory molecules, including CD80 and CD86, and can present antigens to T cells when stimulated with IFN-γ and granulocyte-macrophage colony-stimulating factor (GM-CSF) (20). Toll-like receptor (TLR) signaling has been shown to independently induce costimulatory molecules in neutrophils (20, 21). This so-called antigen-presenting cell (APC)-like phenotype of circulating neutrophils has been identified in acute human sepsis, implying their role in shaping the transition of the innate to the adaptive phase of the antimicrobial immune response (22).

The role of IFN-γ in inducing MHC class II expression in human macrophages and dendritic cells is well established (23, 24). However, the mechanisms underlying this phenotypic shift in response to GM-CSF remain poorly understood. In particular, the signaling pathways leading to an APC-like phenotype in neutrophils during the early phase of human GN bacteremia and sepsis remain scarcely explored. Here, we conducted an in-depth analysis of neutrophils in the early inflammatory phase of human GN bacteremia as a basis for identifying relevant pathomechanisms and potential targets for novel therapeutic approaches.

## Results

### Neutrophils acquire an APC-like phenotype in patients during early Gram-negative bacillary bacteremia

Neutrophils, or polymorphonuclear leukocytes (PMNs), from 23 patients with Gram-negative bacillary bacteremia were isolated at the day of culture-positive diagnosis. The most common infection type was urinary tract infection (74%, n=17), and *Escherichia coli* was the most frequently isolated gram-negative bacterium (74%, n=17). Disease severity was determined according to the recommendations of the Third International Consensus Definitions for Sepsis and Septic Shock (Sepsis-3) (25). Namely, patients with bacteremia/infection and organ disfunction that fulfilled two or more criteria of the quick Sequential Organ Failure Assessment (qSOFA, criteria: systolic blood pressure <100 mmHg, respiratory rate > 22/minute, and altered mental status from baseline) score were considered to have sepsis (i.e., bacteremia with organ dysfunction, 8.7% (n=2) of cases). Patients fulfilling less than two criteria were classified as having infection in 91.3% (n=21). Accordingly, the levels of C-reactive protein (26), a biomarker of inflammation, and procalcitonin (27), a biomarker of bacteremic infection, were elevated compared with normal levels. A total of 10 healthy controls, matched for age and gender, were included (**Table 1**).

**Table 1:** Clinical characteristics of patients and controls.

	Patients	Healthy controls
	n=23	n=10
Demographic characteristics		
Age, years (median, IQR)	74 (69-85)	76 (69-83)
Male (n, %)	13 (57)	6 (60)
Infection type		
Urinary tract (n, %)	17 (74)	–
Pulmonary (n, %)	2 (8.7)	–
Intestinal (n, %)	4 (17.3)	–
Bacterial species		
*Escherichia coli* (n, %)	17 (74)	–
*Pseudomonas aeruginosa* (n, %)	2 (8.7)	–
*Klebsiella oxytoca* (n, %)	1 (4.3)	–
*Klebsiella pneumonia* (n, %)	1 (4.3)	–
*Serratia marcescens* (n, %)	1 (4.3)	–
*Proteus mirabilis* (n, %)	1 (4.3)	–
Severity*		
Infection	21 (91.3)	–
Sepsis	2 (8.7)	–
Septic shock	0 (0)	–
Neutrophil-to-lymphocyte ratio (median, interquartile range)	6.6 (3.3-29.1)	1.4 (1.2-5.0)
C-reactive protein** (median, IQR)	144.8 (118.4-271.8)	–
Pro-calcitonin*** (median, IQR)	0.6 (0.28-37.0)	–
Outcome (day 30 mortality) (n, %)	1 (4.3)	0 (0)

*Sepsis-3 definition with qSOFA score after Singer et al., JAMA 2016.

**CRP is measured in mg/L. Normal values are below 10 mg/L.

***Pro-calcitonin is measured in ng/ml. Normal values are below 0.25 ng/ml.

IQR, interquartile range.

**Figure 1:**
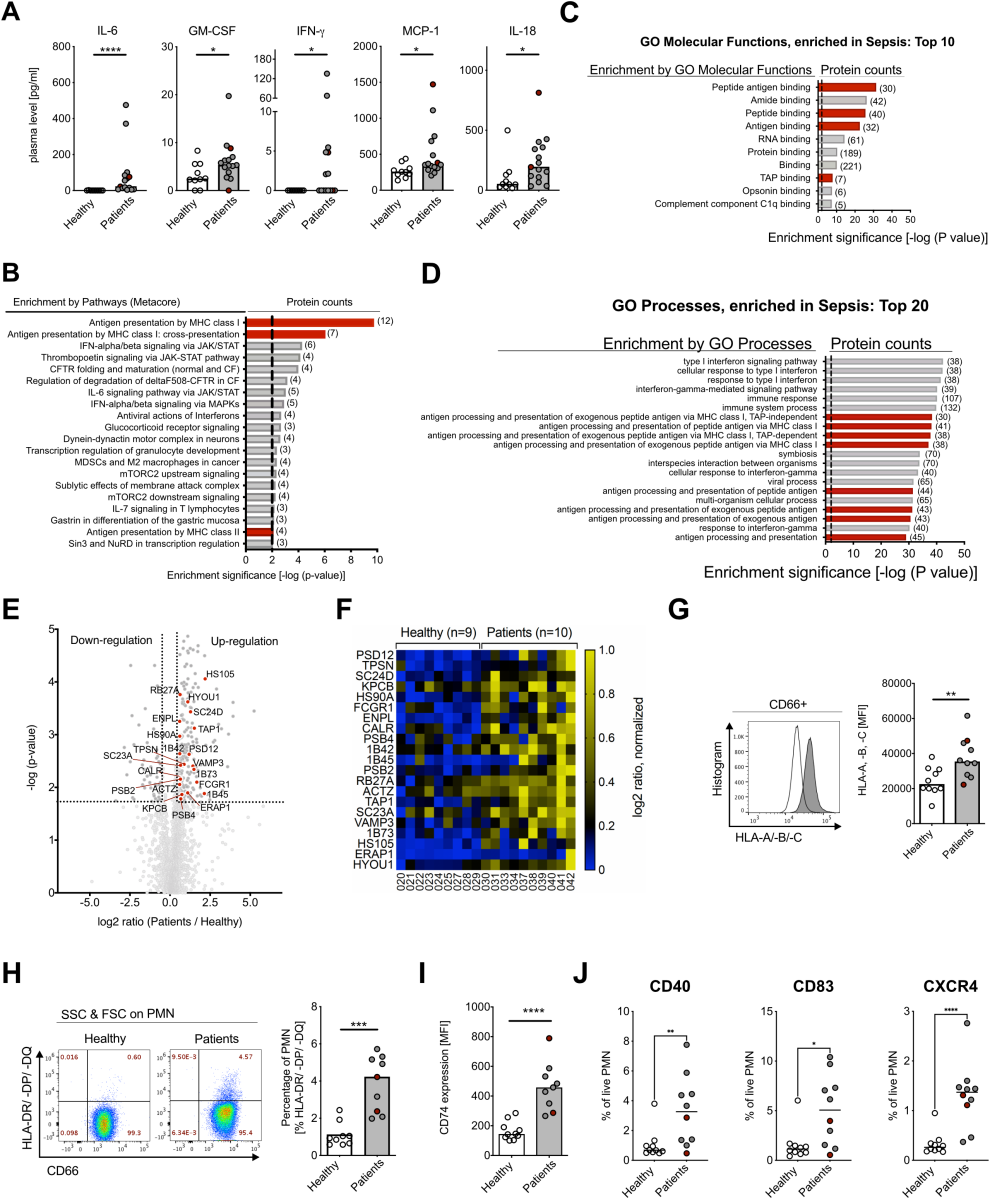
Human neutrophils in gram-negative bacteremia show APC-like phenotype. **(A)** Plasma cytokine concentrations for IL-6, GM-CSF, IFN-γ, MCP-1, and IL-18 of healthy controls and patients. Medians, Mann–Whitney test. **(B)** Enriched pathways from proteomics analysis on neutrophils from healthy controls (n=9) and patients (n=10), using Metacore Enrichment by Pathway Maps. The *P-value* of proteomics data was set < 0.05, threshold = 0, top 20 hits with an enrichment significance of *P-value* < 0.01. Red bars highlight *Antigen Presentation and processing by MHC class I and MHC class II* as highly changed pathways in GN bacteremia patients. **(C)** Gene Ontology (GO) Molecular Functions Enrichment from proteomics analysis, using Metacore Enrichment by GO Molecular Functions. *P-value* of proteomics data was set < 0.05, threshold = 0, top 10 hits with an enrichment significance of *P-value* < 0.01. Red bars highlight Molecular Functions involved in *Antigen processing and presentation* in GN bacteremia. **(D)** GO Process Enrichment from proteomics analysis, using Metacore Enrichment by GO Processes. The *P-value* of proteomics data was set < 0.05, threshold = 0, top 20 hits with an enrichment significance of *P-value* < 0.01. Red bars highlight processes involved in *Antigen processing and presentation* in GN bacteremia. **(E)** Volcano dot plot from proteomics analysis. Highlighted proteins (red dots) are associated with *Antigen presentation and processing by MHC class I and II*, significance threshold (dotted line) was set at *P-value* < 0.02 and fold change ≤ 1.5 cutoff in order of *P-value*. **(F)** Heat map of proteins involved in *Antigen presentation and processing by MHC class I and II*, shown on an individual level with log2 ratio, normalized (0–1, blue to yellow). Significance threshold was set at *P-value* < 0.02 and fold change ≤ 1.5 cutoff in order of *P-value*. Protein names are used according to entry names in UniProt database (www.uniprot.org). **(G)** HLA-A, -B, -C (MHC class I) surface expression on neutrophils of healthy controls and patients, measured with flow cytometry, representative histogram (left) and statistical analysis (right, medians, Mann–Whitney test). **(H)** CD66b and HLA-DR/-DP/-DQ (MHC class II) surface expression on human neutrophils of healthy controls and patients, measured with flow cytometry; representative scatter dot plot (left) and statistical analysis (right, medians, Mann–Whitney test). **(I)** CD74 (Li) surface expression on human neutrophils of healthy controls and patients, measured with flow cytometry. Medians, Mann–Whitney test. **(J)** Co-stimulatory factors of healthy controls and patients, measured with flow cytometry. Medians, Mann–Whitney test. For **(A, G, F),** sepsis patient samples are marked in red. MFI, mean fluorescence intensity. * *P* ≤ 0.05; ** *P* ≤ 0.01, *** *P* ≤ 0.001, **** *P* ≤ 0.0001.

In accordance with previous studies, patients showed significantly increased absolute numbers of circulating neutrophils than healthy controls (**Supplementary Figure S1A**), a significantly higher neutrophil-to-lymphocyte ratio (NLR) (28) (**Supplementary Figure S1B**; **Table 1**), and decreased reactive oxygen species (ROS) production in response to bacterial and fungal stimuli (**Supplementary Figure S1C**). Since infection with gram-negative bacteria is associated with a heterogeneous, inflammatory host response (10, 29), we analyzed blood serum samples from patients and controls for their particular inflammatory cytokine signature. Interleukin (IL)-6, granulocyte macrophage-colony stimulating factor (GM-CSF), interferon-gamma (IFN-γ), monocyte chemotactic protein 1 (MCP-1), and IL-18 were significantly elevated in plasma samples from patients during early Gram-negative bacillary bacteremia (**Figure 1A**), whereas IL-8, IL-1β, IL-17A, IL-23, IL-10, and tumor necrosis factor-alpha (TNF-α) were not changed and IL-12 and IL-33 were not detectable (**Supplementary Figure S1D**).

In order to analyze neutrophil adaptation in bacteremic patients, we performed a systematic proteomics approach of isolated peripheral blood neutrophils. From a total of 2,204 identified peptides, 380 peptides were significantly changed during early GN bacillary bacteremia (**Supplementary Figure S1E**). To identify significant differences in protein expression between cases and controls, unbiased MetaCore-enriched pathway analysis was conducted. Thereby, antigen presentation by MHC classes I and II was identified as strongly enriched in bacteremic patients (**Figure 1B**). This was further confirmed by GO Enrichment Analysis of Processes and Molecular Functions (**Figures 1C, D**). We identified 21 proteins contributing to antigen processing and presentation by MHC class I and MHC class II molecules (30–32) (**Figures 1E, F**), including three MHC class I proteins (1B42, 1B73, 1B45), five ER to Golgi (SC23A, RB27A, HYOU1, SC24D, VAMP3), one molecule in the TAP complex (TAP1), one aminopeptidase (ERAP1), tapasin (TPSN), calreticulin (CALR), one protein of the 26S proteasome complex (PSD12), two proteins of the 20S core proteasome complex (PSB2, PSB4), and others (**Supplementary Figure S1F**).

To verify the proteomics data, we investigated neutrophil surface expression of MHC class I (HLA-A, -B, -C) and class II (HLA-DR/-DP/-DQ^+^) by flow cytometry. Surface staining confirmed a significant increase in MHC class I levels and *de novo* induction of MHC class II expression in bacteremic patients versus controls (**Figures 1G, H**), and the latter was further underpinned by increased surface expression of the invariant chain (CD74) (**Figure 1I**). Part of the MHC class II expressing neutrophils additionally showed expression of co-stimulatory molecules, such as CD40, CD83, and chemokine receptor CXCR4 (**Figure 1J**).In summary, neutrophils during early Gram-negative bacillary bacteremia are in a cytokine-rich, pro-inflammatory environment and show significantly increased absolute numbers of circulating neutrophils, a higher NLR, and lower ROS production. Using an unbiased proteomics approach and flow cytometry, we could demonstrate an APC-like phenotype with elevated MHC class I and *de novo* induced MHC class II expression in neutrophils of patients during early Gram-negative bacteremia.

### APC-like human peripheral blood neutrophils induced *in vitro* by GM-CSF and IFN-γ stimulate T-cell clones upon antigen presentation

Neutrophils are limited in lifespan and difficult to cultivate *ex vivo* (33, 34). Both GM-CSF and IFN-γ have the capacity to reduce neutrophil apoptosis *in vitro* (35, 36). To investigate *de novo* induction of MHC class II on neutrophils and their acquired function, an *in vitro* stimulation model with isolated peripheral blood neutrophils was developed. As peripheral blood neutrophils are very short-lived in culture and rapidly undergo apoptosis, cultures were supplemented with a pan-caspase inhibitor (Q-VD-OPh, or q.OPh) (37), which increases neutrophil life span and allows *in vitro* cultivation of neutrophils for 48 h without significant apoptosis induction (**Supplementary Figures S2A, B**).

**Figure 2:**
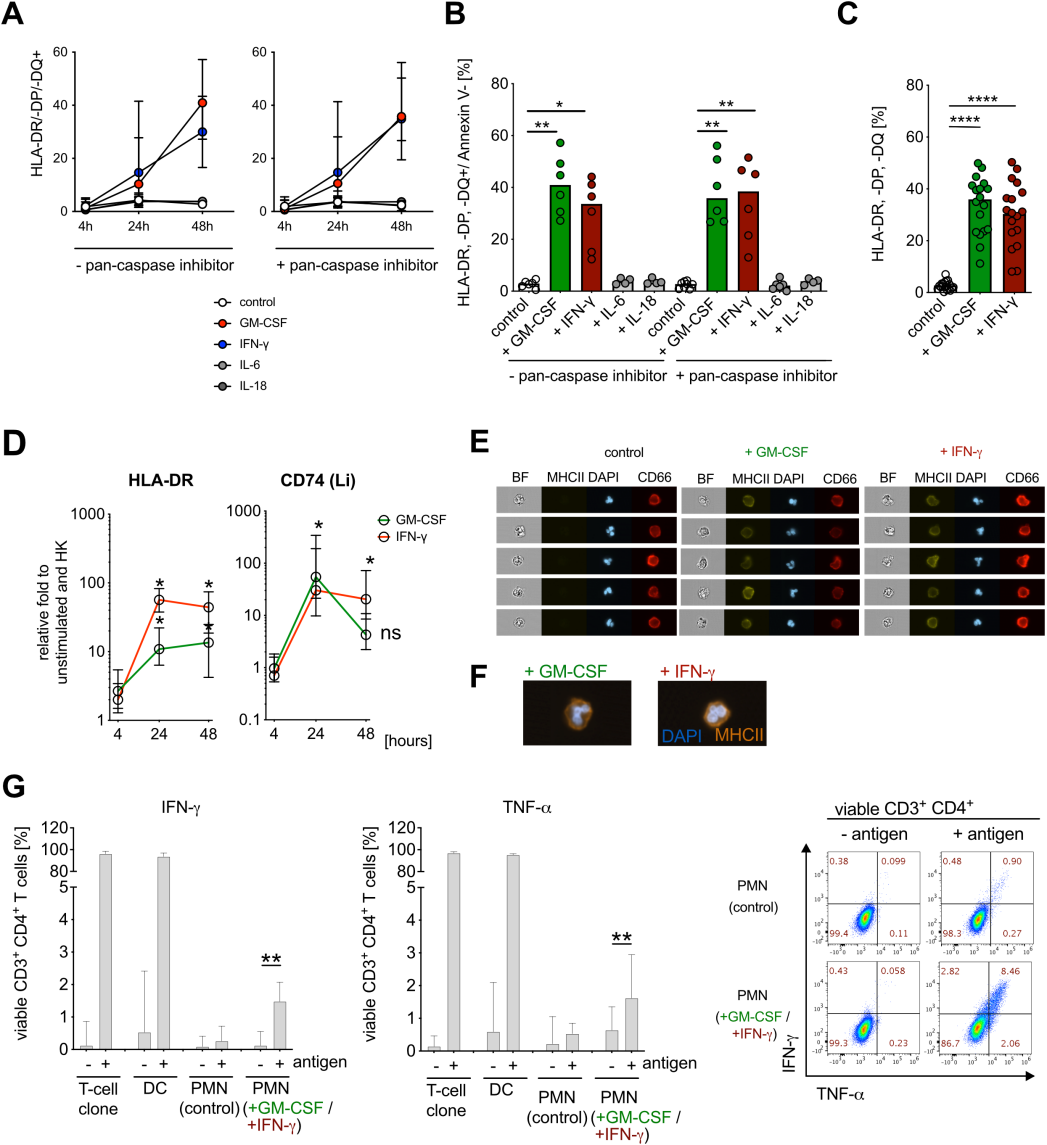
IFN-γ and GM-CSF induce APC-like neutrophil phenotype *in vitro*. **(A)** HLA-DR/-DP/-DQ (MHC class II) surface expression on human neutrophils (CD66b^+^ cells, gated on Annexin V^−^) after stimulation with human recombinant GM-CSF (10 ng/ml), IFN-γ (10 ng/ml), IL-6 (10 ng/ml) and IL-18 (10 ng/ml) for 48 h, preincubated −/+ pan-caspase inhibitor q.OPh (3 µM) for 4 h, 24 h, and 48 h, measured consequently with flow cytometry, medians with range (n=6). **(B)** Summary of HLA-DR/-DP/-DQ^+^/Annexin V^−^ neutrophils after pre-incubation with −/+ q.OPH and stimulation with GM-CSF, IFN-γ, IL-6, and IL-18 for 48h. Medians, Kruskal–Wallis test (compared with controls). **(C)** Percentage of HLA-DR/-DP/-DQ^+^/Annexin V^−^ neutrophils after pre-incubation with q.OPh and stimulation with GM-CSF and IFN-γ for 48h. Medians (n=18), Mann–Whitney test. **(D)** HLA-DR and Li (CD74) mRNA expression after stimulation with GM-CSF and IFN-γ for 4 h, 24 h, and 48h. Values are shown as relative fold change to unstimulated control and internal control (housekeeping genes, HK) (medians, n=6, Mann–Whitney test; ns, not significant). **(E)** *de novo* MHC class II (HLA-DR/-DP/-DQ) surface molecule expression on neutrophils after stimulation with GM-CSF and IFN-γ for 48 h, measured with ImageStreamX, representative example. DAPI was used for nuclear staining and CD66b as a surface expression marker. BF, bright field. **(F)** Representative overlay of *de novo* MHC class II (HLA-DR/-DP/-DQ, orange) surface molecule expression on neutrophils after stimulation with GM-CSF and IFN-γ for 48 h, measured by ImageStreamX at 60× magnification. DAPI (blue) was used for nuclear staining and CD66b as a surface expression marker. **(G)** Intracellular cytokine staining for IFN-γ- and TNF-α-positive viable CD3^+^ CD4^+^ T cells, co-incubated with PMN or antigen-pulsed PMN [PMN (control), ratio 1:10], and cytokine-stimulated PMN [GM-CSF (10 ng/ml) + IFN-γ (10 ng/ml)] or antigen-pulsed cytokine-stimulated PMN (ratio 1:10) for 6h. Percentage for IFN-γ and TNF-α expression (left), and representative scatter dot plot (right). As a positive control, autologous dendritic cells (DCs, 1:1 ratio) and T-cell clones alone were used. All values are shown as medians with interquartile ranges (n=11; n=6 for positive control with DCs; Wilcoxon signed rank test; * *P* ≤ 0.05; ** *P* ≤ 0.01, **** *P* ≤ 0.0001; ns, not significant.

First, we investigated the ability of cytokines detected during early bacteremia to induce an APC-like phenotype. GM-CSF and IFN-γ were both able to induce *de novo* MHC class II expression (**Figures 2A–C**), whereas IL-6 and IL-18 had no effect (**Figures 2A, B**). Addition of the pan-caspase inhibitor had no impact on MHC class II expression (**Figures 2A, B**; **Supplementary Figure S2A**). *De novo* induction of HLA-DR and CD74 (Li) by GM-CSF and IFN-γ stimulation was further confirmed on RNA level (**Figure 2D**). Interestingly, while stimulation with IFN-γ resulted in the elevation of MHC-class I expression, this effect was not observed upon stimulation with other cytokines, including GM-CSF (**Supplementary Figures S2C, D**).

Previous reports described an APC-like phenotype mostly in immature neutrophil populations (38). To assess the maturation stage of MHC class II expressing neutrophils induced by GM-CSF and IFN-γ stimulation of peripheral blood neutrophils, we visualized single cells by imaging flow cytometry. We found that HLA-DR/-DP/-DQ expression co-localizes with the surface marker CD66b and that HLA-DR/-DP/-DQ+ neutrophils exhibit segmented nuclear morphology, suggesting that neutrophils expressing APC-like molecules are mature cells (39) (**Figures 2E, F**, gating strategy **Supplementary Figure S2E**).

We next wanted to know whether APC-like neutrophils can stimulate T cells. We stimulated neutrophils with GM-CSF and IFN-γ for 48 h to induce an APC-like phenotype, pulsed them with specific peptide, and demonstrated activation of an autologous peptide-specific CD4^+^ T-cell clone by intracellular cytokine staining for IFN-γ and TNF-α (40) (**Figure 2G**). APC-like neutrophils activated CD4+ T cells *in vitro*, although this is a minor effect compared with professional APCs such as DCs, which might be due to insufficient expression of co-stimulatory molecules (as in **Figure 1J**).

Taken together, GM-CSF and IFN-γ induce *de novo* expression of MHC class II on mature peripheral blood neutrophils *in vitro*, which enables autologous CD4+ T-cell activation *in vitro* at moderate levels by these APC-like neutrophils.

### GM-CSF induced MHC class II expression on neutrophils is tightly regulated by JAK1/2 signaling

**Figure 3:**
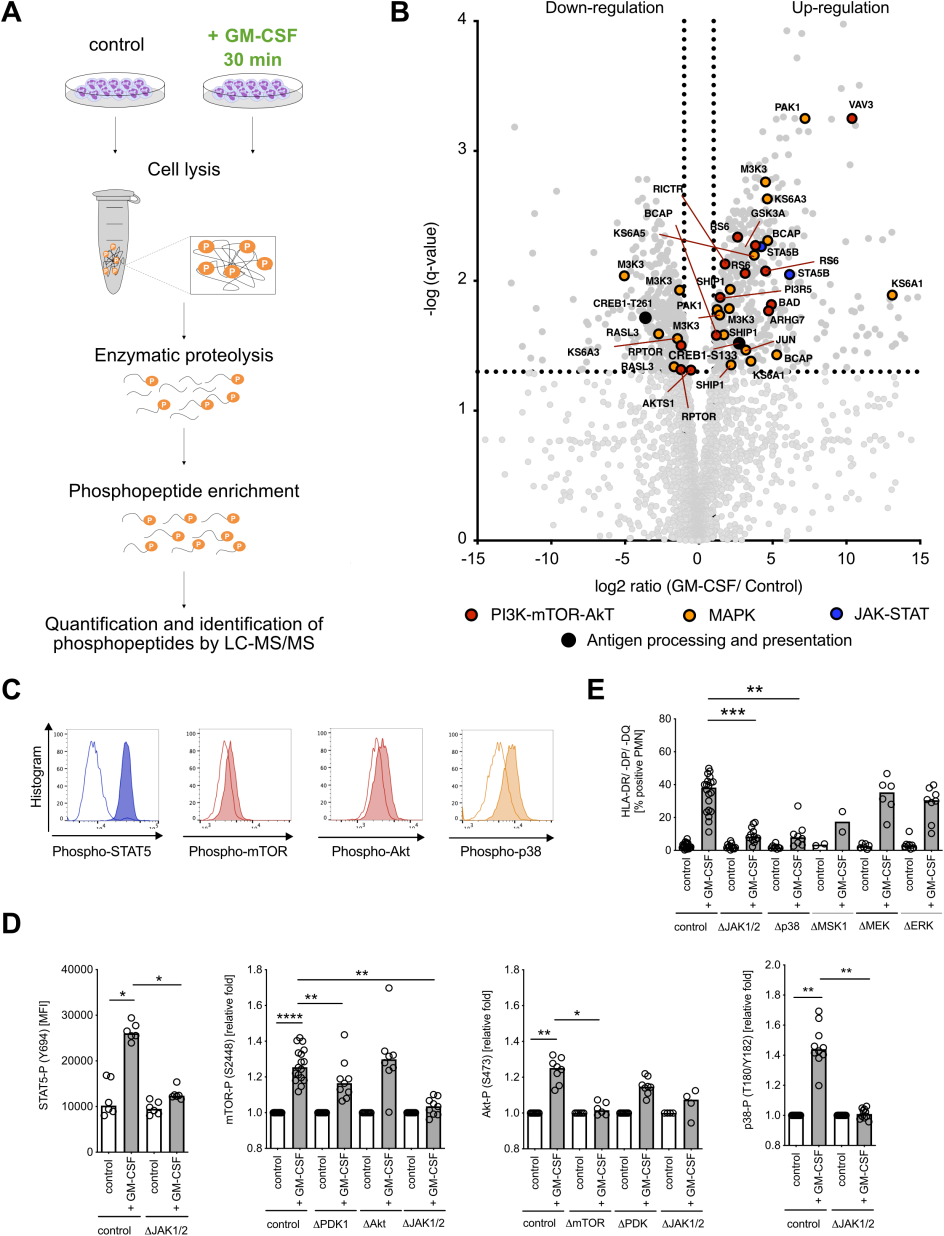
GM-CSF signaling leads to the activation of JAK-STAT, MAPK p38, and mTOR-Akt signaling pathways and phosphorylation of transcription factor CREB. **(A)** Visualization of the phosphoproteomics experimental procedure. Neutrophils (10^8^ cells each condition) were stimulated with GM-CSF (10 ng/ml) for 30 min and then collected for further processing. **(B)** Volcano dot plot of detected phosphopeptide changes (n=3,579) after stimulation with GM-CSF for 30 min. The significance threshold was set at *q-value* < 0.05 and fold change ≤ 2 cutoff in order of *P-value*. **(C)** Representative histograms for STAT5- (Y694), mTOR- (S2448), Akt- (S473), and p38- (T180/Y182) phosphorylation after GM-CSF stimulation for 30 min by using Phosflow antibodies for flow cytometry. Unstimulated control is shown in white, GM-CSF stimulation is shown shaded. **(D)** STAT5- (Y694), mTOR- (S2448), Akt- (S473), and p38- (T180/Y182) phosphorylation after GM-CSF stimulation, measured by phospho flow cytometry. Inhibitors ruxolitinib (ΔJAK1/2 inhibitor, final conc. = 3 µM), BX-795 (ΔPDK1 inhibitor, final conc. = 10 µM), MK-2206 (ΔAkt1/2/3 inhibitor, final conc. = 10 µM), and PP242 (ΔmTORC1/2 inhibitor, final concentrations = 5 µM) were used. Medians, Kruskal–Wallis test. **(E)** HLA-DR/-DP/-DQ^+^ (MHC class II^+^)/Annexin V^−^ neutrophils after pre-incubation with/without inhibitors ruxolitinib (ΔJAK1/2 inhibitor, final concentrations = 3 µM), SB203580 (Δp38 inhibitor, final conc. = 10 µM), SB 747651 A (ΔMSK1 inhibitor, final conc. = 10 µM), trametinib (ΔMEK1/2 inhibitor, final conc. = 10 µM), and SCH772984 (ΔERK1/2 inhibitor, final conc. = 5 µM), and stimulation with human recombinant GM-CSF (10 ng/ml) for 48h. Medians, Kruskal–Wallis test. MFI, mean fluorescence intensity. * *P* ≤ 0.05; ** *P* ≤ 0.01, **** *P* ≤ 0.0001.

We next examined the signaling pathways responsible for cytokine-induced MHC class II expression in neutrophils. Since the IFN-γ signaling pathway associated with MHC class II induction is already well described in human vascular endothelial cells and macrophages (23, 24), we focused on GM-CSF-induced MHC class II expression. We used a label-free quantitative phosphoproteomics strategy and analyzed phosphorylation by liquid chromatography-tandem mass spectrometry (41) (**Figure 3A**). We detected a total of 3,579 phosphopeptides in GM-CSF-stimulated human neutrophils. Of these, 858 phosphopeptides showed a significantly changed phosphorylation status compared with unstimulated cells (466 phosphopeptides showed induced phosphorylation, 392 showed dephosphorylation, q-value < 0.05). The majority of significantly changed phosphorylation and dephosphorylation events were identified on serine and threonine residues (**Supplementary Figure S3A**). The phosphoproteomics data set revealed the induction of mitogen-activated protein kinase (MAPK), Janus kinase–signal transducer and activator of transcription (JAK-STAT), and phosphatidylinositol-3-kinase–protein kinase B–mammalian target of rapamycin (PI3K-Akt-mTOR) signaling pathways after GM-CSF stimulation *in vitro*, which is in agreement with literature (**Figure 3B**) (42). We confirmed the involvement of the identified pathways by phospho flow cytometry using antibodies against phospho-STAT5 (pY694), phospho-mTOR (pS2448), phospho-Akt (pS473), and phospho-p38 (pT180/pY182) (**Figure 3C**) as well as by pharmacological inhibition of JAK1/2 (ruxolitinib), PDK-1 (BX-745), Akt (MK-2206), and mTORC1/2 (PP242) (**Figure 3D**). JAK1/2 inhibition with ruxolitinib led to full inhibition of STAT5, mTOR, and p38 phosphorylation in human neutrophils, whereas inhibition of mTOR led to inhibition of Akt. Furthermore, JAK1/2 inhibition as well as p38 inhibition nearly abolished GM-CSF-induced MHC class II expression in neutrophils (**Figure 3E**). We identified an important candidate linking GM-CSF signaling to APC-like phenotype, cAMP-responsive element binding protein 1 (CREB1, **Supplementary Figure S3B**), whose phosphorylation consequently leads to MHC class II gene expression (43). This pathway is investigated in detail in the next chapter.

Taken together, GM-CSF stimulation in neutrophils affects three major signaling pathways, the JAK-STAT, MAPK, and PI3K-Akt-mTOR pathways (**Supplementary Figure S3B**), with a central JAK1/2 kinase orchestrating broad downstream protein phosphorylation ultimately leading to MHC class II induction.

### GM-CSF stimulation induces the MHC class II enhanceosome in human neutrophils

**Figure 4:**
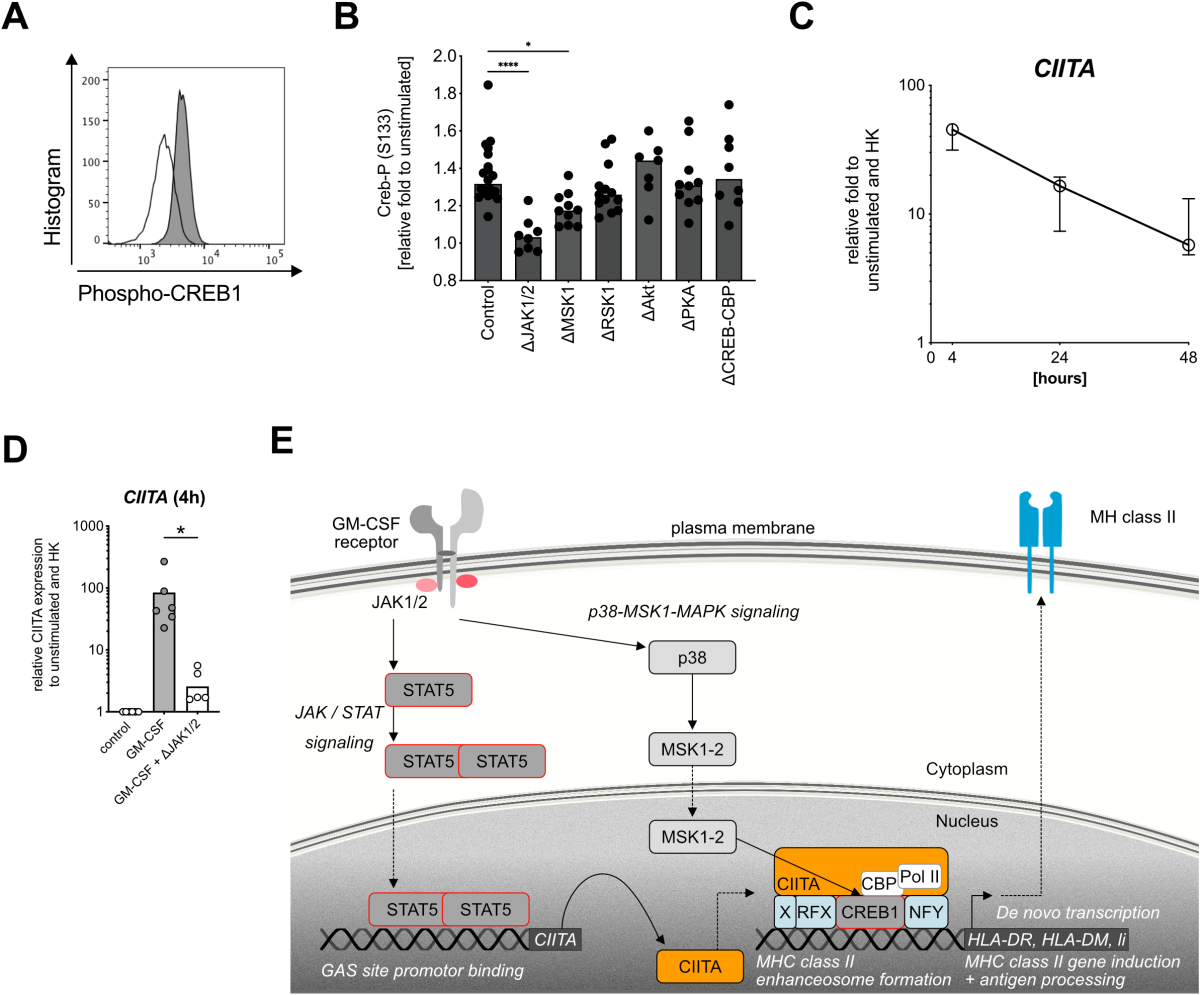
Targeting the MHC class II enhanceosome in human neutrophils. **(A)** Representative histogram for CREB1 phosphorylation (S133) in healthy donor neutrophils after GM-CSF stimulation (gray) compared with unstimulated (white) for 30 min by using Phosflow antibody for flow cytometry. **(B)** CREB1 phosphorylation at S133 (Creb-P) after GM-CSF stimulation, measured by phospho flow cytometry, relative fold changes to unstimulated control. Medians, Kruskal–Wallis test. Inhibitors ruxolitinib (ΔJAK1/2 inhibitor, final conc. = 3 µM), SB 747651 A (ΔMSK1 inhibitor, final conc. = 10 µM), BI-D1870 (ΔRSK1-4 inhibitor, final conc. = 10 µM), and MK-2206 (ΔAkt1/2/3 inhibitor, final conc. = 10 µM), CAS 92-78-4 (ΔCREB-CBP interaction inhibitor, final conc. = 50 µM) were used. **(C)** *CIITA* mRNA expression after stimulation with human recombinant GM-CSF (10 ng/ml) for 4 h, 24 h, and 48h. Values are shown as relative fold change to unstimulated control and internal control (housekeeping genes, HK). Medians with interquartile range, n=6. **(D)** CIITA mRNA expression after stimulation with human recombinant GM-CSF for 4 h, pretreated with or without ruxolitinib for 1h. Relative control to unstimulated and housekeeping genes (HK). Medians, comparison of GM-CSF-stimulated via Wilcoxon signed rank test. **(E)** Human neutrophil signaling cascade leading to *de novo* MHC class II induction after stimulation with GM-CSF. JAK1/2, Janus kinase 1 and 2; STAT5, signal transducer and activator of transcription 5; Y694, tyrosine phosphorylation site at residue 694; CIITA, MHC class II transactivator; p38, p38 mitogen-activated protein kinase; MSK1, mitogen- and stress-activated protein kinase-1; RFX, regulatory factor X; CREB1, cAMP-responsive element binding protein 1; Pol II; polymerase II; CBP, CREB-binding protein; HLA-DR, HLA-DM, Li, MHC class II genes. * *P* ≤ 0.05; **** *P* ≤ 0.0001.

*De novo* MHC class II induction in other non-professional APC such as human vascular endothelial cells and macrophages was shown to be dependent on the formation of the MHC class II enhanceosome (23, 24). In these cell types, cyclic AMP response element-binding protein1 (CREB1) (24, 44) phosphorylation is an essential part in the formation of the MHC class II enhanceosome (43). Indeed, our phosphoproteomics data similarly showed a significant increase of CREB1 phosphorylation at Serine 133 in GM-CSF-stimulated neutrophils (**Figure 3B**), which we could further confirm by phospho flow cytometry (**Figure 4A**). Potential candidates for CREB1 phosphorylation include such kinases as MSK1 (45), RSK (46), AKT (47), and PKA (48). Using pharmacological inhibitors specifically targeting these kinases, we could demonstrate that only MSK1 inhibition significantly reduced CREB1 phosphorylation (**Figure 4B**). Inhibition of the interaction between CREB-1 and CREB-binding protein (CBP) did not affect CREB1 phosphorylation either (**Figure 4B**); however, it significantly reduced HLA-DR/-DP/-DQ/surface expression (**Supplementary Figure S4A**).

In addition to CREB1 phosphorylation, the transcriptional control of MHC class II gene expression in other cell types is tightly regulated by other three key factors [reviewed in (24)], namely, the MHC class II transactivator CIITA (49, 50), NFY (51), and the RFX complex composed of RFX5 (52), RFXAP (53), and RFXANK (54). To investigate whether *de novo* MHC class II expression in neutrophils uses a similar mechanism, we analyzed CIITA mRNA levels after GM-CSF stimulation and found a 100-fold CIITA upregulation after 4 h, with a gradual decrease over time (**Figure 4C**). CIITA induction could be fully blocked by using a JAK1/2 inhibitor (**Figure 4D**), again highlighting the central role of JAK1/2 signaling for MHC class II induction in neutrophils. In agreement with literature, NFYa, RFXank, and CREB mRNA levels were not affected by GM-CSF stimulation (**Supplementary Figure S4B**), confirming CIITA as the master transcriptional regulator of the MHC class II enhanceosome (24, 50).

To summarize, we demonstrated that GM-CSF-induced *de novo* expression of MHC class II in neutrophils is mediated by the same MHC class II enhanceosome that also regulates IFN-γ-induced, non-constitutive MHC class II expression in human vascular endothelial cells and macrophages (23). GM-CSF-induced MHC class II induction is mediated by a MAPK-p38-MSK1-CREB1 signaling cascade and the MHC class II transactivator CIITA in a strictly JAK1/2 kinase-dependent manner (**Figure 4E**).

## Discussion

Neutrophils in bacteremia and sepsis are believed to execute direct pathogen killing (55). In this study, we identified a subset of neutrophils with antigen-presenting properties during the hyperinflammatory phase of sepsis. These MHC class II^+^ neutrophils can be induced by GM-CSF and IFN-γ via the formation of the MHC class II enhanceosome and are able to present antigens and activate peptide-specific T-cell clones. Thus, the role of neutrophils in bacteremia/sepsis seems to go beyond direct pathogen killing mechanisms and is pointing toward immune-regulatory functions.

Moderate levels of MHC class II on neutrophils have been previously identified in human autoimmune diseases such as granulomatosis with polyangiitis (56, 57), atherosclerosis (58), and rheumatoid arthritis (59). However, it is controversially discussed whether similar mechanisms exist in the context of bacterial infections (60–62). An immature Gr-1^+^CD11b^+^ neutrophil population in a murine mouse model of polymicrobial sepsis was found, which contributed to sepsis-induced T-cell suppression and T_H_2 polarization (63). In our study, we found that during the early phase of sepsis, neutrophils acquire antigen-presenting characteristics. Simultaneously high levels of IFN-γ and GM-CSF levels were detected in plasma of the sepsis patients. Similarly as previously shown (56, 64–66), both cytokines can induce *de novo* MHC class II transcription and surface expression on human neutrophils. Thus, we propose that neutrophils acquire novel characteristics associated with antigen processing and presentation under infectious conditions and oblige inflammation, and not bacterial infection *per se*, as a key factor for the induction of APC-like neutrophils.

While previous studies focused on proteomic changes of secreted proteins from *ex vivo* stimulated neutrophils with cytoB/fMLF from sepsis patients (67), this is to our knowledge the first study comprehensively investigating the proteomic changes in human neutrophils during GN-bacteremia.

Our work adds to studies showing that MHC class II and costimulatory molecules are induced in the presence of antigen and antigen-specific memory CD4^+^ T cells (20). Most likely, antigen-specific memory CD4^+^ T-cell derived cytokines such as GM-CSF and IFN-γ induced the observed MHC class II expression on neutrophils *in vitro*, as we demonstrated in our study. Supporting this idea, we could observe that antigen-specific CD4^+^ T-cell clones produce large amounts of TNF-α and IFN-γ upon activation (68).

We further investigated signaling mechanisms that control *de novo* MHC class II expression in neutrophils. Previous studies could show that the control of *de novo* MHC class II expression in various cell types is tightly regulated by several key factors (reviewed in (24)), namely, the MHC class II transactivator CIITA (49, 50), CREB1 (43), NFY (51), and the RFX complex (composed of RFX5 (52), RFXAP (53), and RFXANK (54)), and that genetic deficiencies in those key factors can cause severe pathologies such bare lymphocyte syndrome (BLS) (49) and lymphoid cancers (69). However, since these studies on *de novo* MHC class II induction were restricted to the IFN-γ signaling pathway in human vascular endothelial cells and macrophages (23, 24, 70), it was vastly unclear how GM-CSF-mediated signaling factors were related to the induction of MHC class II molecules in different cell types. In this study, we found that GM-CSF phosphorylates CREB1 at Ser133 *in vitro* and that CREB1-CBP protein interaction inhibition strongly decreases *de novo* HLA-DR/-DP/-DQ expression on neutrophil surface. Furthermore, we could demonstrate that GM-CSF-mediated signaling leads to the induction of CIITA transcription, similarly to IFN-γ signaling, whereas NFY and RFX levels are not affected. Conclusively, our data highlight for the first time a major role of GM-CSF-induced CREB1 phosphorylation and CIITA induction in APC-like neutrophils.

CREB1 is a substrate for various cellular kinases (71) such as MSK1 (45), pp90^RSK^ (or RSK2) (46), AKT (47), PKA (48), and MAPKAP-2 (72). By using pharmacological inhibitors specifically targeting these kinases, we demonstrated that MSK1, but not pp90^RSK^, AKT, and PKA pp90^RSK^, AKT, and PKA inhibition significantly reduces CREB1 phosphorylation. We therefore strongly support the role of the MAPK-p38-MSK1-CREB1 signaling axis as a driver for *de novo* MHC class II induction on human neutrophils. Another group could show that LPS or TNF-stimulated human neutrophils led to cytokine production involving the p38-MSK1-CREB1 axis (73). Therefore, we postulate that CREB1 is an important transcription factor that regulates a vast repertoire of immune responses in neutrophils involved in inflammatory processes.

It is also known that IFN-γ mediates induction of MHC class II presentation through JAK-STAT signaling (24). In the present study, we demonstrated that JAK1/2 inhibition with ruxolitinib at non-toxic concentrations led to full inhibition of GM-CSF-mediated STAT5, mTOR, Akt, p38, and CREB1 phosphorylation in human neutrophils, rendering JAK1/2 an interesting drug target in neutrophils to cease a huge variety of signaling pathways at once (74), including those signaling events leading to the APC-like phenotype. So far, the clinical use of JAK1/2 inhibitors is approved in patients suffering from myelofibrosis and rheumatoid arthritis and they are currently in the clinical development targeting several indications such as graft-versus-host-disease, pancreatic cancer, and myeloproliferative diseases (75). It remains to be elucidated whether JAK1/2 inhibitors may be useful as a treatment option during the course of sepsis (76).

HLA-DR/-DP/-DQ surface molecule expression on neutrophils co-localizes with CD66b, a specific marker consistently expressed on human neutrophil surface independent of the cell location, level of activation, and disease state (77). Interestingly, nuclear staining revealed that those HLA-DR/-DP/-DQ^+^ neutrophils show a segmented nuclei morphology, classically known for mature neutrophils (39). This finding is contrary to a report showing that APC-like hybrid tumor-associated neutrophils (TANs) in early-stage lung cancer exhibit characteristics of neutrophils and antigen-presenting cells with a round, immature cell morphology (38). These discrepancies in relation to nuclear morphology could be partially explained by the various key factors involved in extravasation and the local tumor microenvironment that may influence the neutrophil nuclear phenotype.

Lastly, while we observed elevated MHC class I levels on PMN from bacteremic patients compared with healthy donors, this effect was shown *in vitro* only upon stimulation of neutrophils by IFN-γ and not by GM-CSF. It was previously demonstrated that neutrophils can present bacterial antigens through MHC class I (78). This may be a reason for the observed upregulation in patient neutrophils, and the MHC class I upregulation by *in vitro* stimulation suggests that IFN-γ might be a direct contributor.

Classical APCs such as DC and macrophages orchestrate adaptive immune response by T-cell activation. Our data show that APC-like neutrophils can present peptides and activate autologous CD4^+^ T-cell clones *in vitro*. However, T-cell activation by APC-like neutrophils is moderate compared with professional APCs such as DC (20). Therefore, the role of neutrophils as APCs should be considered with certain caveats. While our data show that neutrophils can express MHC class II and the invariant chain (CD74), which are essential for antigen presentation, the extent to which these cells can process and present antigens effectively remains an open question. Additionally, the functional relevance of MHC class II expression in neutrophils during infections and how it impacts adaptive immune responses are areas that require further exploration. Understanding these aspects will provide a more comprehensive view of how neutrophils contribute to the immune response during infection, beyond their traditional roles in phagocytosis and microbial killing.

In conclusion, neutrophils can express features of atypical APCs by upregulating MHC class II expression during GN bacteremia. A comprehensive phosphoproteomics analysis of mature neutrophils stimulated *in vitro* with GM-CSF revealed the induction of the JAK-1 STAT, MAPK, and PI3K-Akt-mTOR pathways. This process leads to the formation of the MHC class II enhanceosome via CREB1 phosphorylation at Serine133 and *de novo* induction of the MHC class II transactivator CIITA, with a central role for JAK1/2 kinase. Targeting this pathway, e.g., with ruxolitinib, could have a significant impact on the development of new therapies in GN bacteremia and sepsis.

## Materials and methods

### Patients and healthy volunteers

The single-center prospective clinical study was performed at the University Hospital Basel, Switzerland. In total, 34 participants with confirmed gram-negative bacteremia (two or more diagnostic criteria for systemic inflammatory response syndrome (SIRS) (79) plus confirmed presence of gram-negative bacteria in blood culture, n=24) and healthy controls (n=10) matching for age and body mass index were recruited between June and November of 2016. Exclusion criteria for patients and controls were pregnancy and lipid disorders. One patient was excluded due to technical reasons (P6). The study was approved by the ethical committee of Nordwest- and Zentralschweiz (BASEC Project ID: 2016-00676) and was in agreement with the Declaration of Helsinki and Good Clinical Practice (GCP) Guidelines.

Patient severity was determined according to the recommendations of the Third International Consensus Definitions for Sepsis and Septic Shock (Sepsis-3) (25). Namely, patients with bacteremia/infection that fulfilled two or more criteria of the quick Sequential Organ Failure Assessment (qSOFA) score were considered to possibly have sepsis (i.e., bacteremia plus organ dysfunction). Patients with bacteremia receiving vasopressor therapy required to maintain mean arterial pressure above 65 mmHg and lactate levels >2 mmol/L despite adequate fluid resuscitation were considered to have septic shock. Peripheral blood was drawn within 24 h of confirmed bacteremia. Plasma was obtained by centrifugation (10 min, 1,600 × g).

### Human PMN isolation

Human PMNs were isolated as previously described (80, 81). In brief, human peripheral blood was collected in 7.5-ml polyethylene tubes containing 1.6 mg EDTA/ml blood (Sarstedt), mixed with 3% Dextran (Pharmacia)/NaCl solution. The leukocyte-rich plasma was transferred to a discontinuous Percoll gradient with 53% and 67% Percoll (GE Healthcare). Percoll gradient centrifugation was performed for 30 min at 1,400 rpm, 4°C, no braking. The visible ring containing the PMN fraction was collected and washed in 0.9% NaCl, resuspended in RPMI (Invitrogen Gibco) + 10% FBS, respectively 10% HS. Cells were counted with Türk’s solution and an automatic cell counter system ADAM (Digital Bio). Purity and viability was routinely >97% and >99%, respectively.

### Pathogen cultures

*Salmonella* strains used in this study were derived from *Salmonella enterica* serovar Typhimurium *SL1344 hisG rpsL xyl* (82, 83). *Salmonella* were cultured at 37°C with aeration (200 rpm) in Lennox LB. *Salmonella* were grown to mid-log phase, washed twice in PBS, and used for *in vivo* experiments. Heat inactivation was performed at 99°C for 15 min. Heat-inactivated *Salmonella* were opsonized in 10% human serum in PBS for 20 min at 37°C, washed with PBS, and diluted to MOI 200 for immediate use (heat-inactivated *Salmonella*).

*Candida albicans* SC5314 was grown overnight in yeast peptone dextrose (YPD, BD Difco) media at 37°C as previously described (81). A subculture was inoculated 1:100 and grown to mid-log phase. *C. albicans* was washed twice with 0.9% NaCl and heat-inactivated at 95°C for 1 h. *C. albicans* was opsonized in 10% human serum in PBS for 20 min at 37°C, washed with PBS, and diluted to MOI 1 for immediate use.

### Inhibitors and cytokines

Ruxolitinib (JAK1/2 inhibitor, Cat. No. S1378), PD98059 (MEK1 inhibitor, Cat. No. S1177), trametinib (MEK1/2 inhibitor, Cat. No. S2673), wortmannin (PI3K inhibitor, Cat. No. S2758), neratinib (EGFR1/2 inhibitor, Cat. No. S2150), LY294002 (PI3K inhibitor, Cat. No. S1105), IPA-3 (PAK1 inhibitor, Cat. No. S7093), BX-795 (PDK-1 inhibitor, Cat. No. S1274), MK-2206 (Akt1/2/3 inhibitor, Cat. No. S1078), BI-D1870 (RSK1-4 inhibitor, Cat. No. S2843), SB203580 (p38 MAPK inhibitor, Cat. No. S1076), SCH772984 (ERK1/2 inhibitor, Cat. No. S7101), H89 (PKA inhibitor, Cat. No. S1582), PP242 (mTORC1/2 inhibitor, Cat. No. S2218), and rapamycin (mTORC1 inhibitor, Cat. No. S1039) were obtained from Selleckchem. SB747651A (MSK1 inhibitor, Cat. No. 4630) was obtained from Tocris. CAS 92-78-4 (CREB-CBP inhibitor, Cat. No. 217505) was obtained from Calbiochem. Human recombinant GM-CSF, IFN-γ, IL-6, and IL-18 were ordered by PeproTech.

### Sample preparation for proteomics (LC-MS) analysis

5 × 10^5^ freshly isolated human neutrophils were used for proteomics analysis. Cells were washed twice with PBS (Sigma) and were lysed in 200 μl lysis buffer (2% sodium deoxycholate (SDC), 0.1 M ammonium bicarbonate) using strong ultrasonication (two cycles of sonication S3 for 10 s, Hielscher ultrasonicator). The protein concentration was determined by BCA assay (Thermo Fisher Scientific) using a small sample aliquot. 50 μg of proteins was digested as described previously (84), reduced with 5 mM TCEP for 15 min at 95°C, and alkylated with 10 mM iodoacetamide for 30 min in the dark at 25°C. After diluting samples with 100 mM ammonium bicarbonate buffer to a final DOC concentration of 1%, proteins were digested by incubation with sequencing-grade modified trypsin (1/50, w/w; Promega, Madison, Wisconsin) overnight at 37°C. Then, the samples were acidified with 2 M HCl to a final concentration of 50 mM, incubated for 15 min at 37°C and the precipitated detergent removed by centrifugation at 10,000×g for 15 min. Subsequently, peptides were desalted on C18 reversed-phase spin columns according to the manufacturer’s instructions (MicroSpin, Harvard Apparatus) and dried under vacuum.

### TMT labeling and HpH fractionation

The dried peptide samples were subsequently labeled with isobaric tag (TMT10plex, Thermo Fisher Scientific) according to the manufacturer’s instructions. To control for ratio distortion during quantification, a peptide calibration mixture consisting of six digested standard proteins mixed in different amounts was added to each sample before TMT labeling as described (84). After pooling the TMT-labeled peptide samples, peptides were again desalted on C18 reversed-phase spin columns according to the manufacturer’s instructions (Macrospin, Harvard Apparatus) and dried under vacuum. TMT-labeled peptides were fractionated by high-pH reversed phase separation using an XBridge Peptide BEH C18 column (3,5 µm, 130 Å, 1 mm × 150 mm, Waters) on an Agilent 1260 Infinity HPLC system. Peptides were loaded on column in buffer A (ammonium formate (20 mM, pH 10) in water) and eluted using a two-step linear gradient starting from 2% to 10% in 5 min and then to 50% (v/v) buffer B (90% acetonitrile/10% ammonium formate (20 mM, pH 10) over 55 min at a flow rate of 42 µl/min. Elution of peptides was monitored with a UV detector (215 nm, 254 nm). A total of 36 fractions were collected, pooled into 12 fractions using a post-concatenation strategy as previously described (85), dried under vacuum, and subjected to LC-MS/MS analysis.

### Sample preparation for phosphoproteomic analysis

Samples were prepared as previously described (41). In brief, for each condition, 10^8^ neutrophils were stimulated for 30 min without or with GM-CSF. Next, the cells were put on ice and washed twice with ice-cold phosphate-buffered saline (PBS). Samples were collected in urea solution (8 M urea, AppliChem, Darmstadt, Germany), 0.1 M ammonium bicarbonate (Sigma, St. Louis, MO), 0.1% RapiGest (Waters, Milford, MA) in the presence of phosphatase inhibitor 1× PhosSTOP (Roche, Basel, Switzerland). Supernatants were collected and stored at −80°C for further processing. BCA Protein Assay (Pierce, Rockford, IL) was used to measure protein concentration.

### Phosphopeptide enrichment for phosphoproteomics

2 mg of total protein lysate was digested with trypsin, cleaned up using an C18 column, and enriched for phosphorylated peptides using titanium dioxide beads as described (41). After C18 cleanup, 1 µg of peptides was LC-MS analyzed as described below with the following changes: the normalized collision energy was set to 27%, and the mass isolation window was set to 1.4 m/z. The acquired raw files were imported into the Progenesis QI software (v2.0, Nonlinear Dynamics Limited), which was used to extract peptide precursor ion intensities across all samples applying the default parameters. The generated mgf files were searched using MASCOT as above using the following search criteria: full tryptic specificity was required (cleavage after lysine or arginine residues, unless followed by proline); three missed cleavages were allowed; carbamidomethylation (C) was set as fixed modification; oxidation (M) and phosphorylation (STY) were applied as variable modifications; mass tolerance of 10 ppm (precursor) and 0.02 Da (fragments). The database search results were filtered using the ion score to set the false discovery rate (FDR) to 1% on the peptide and protein level, respectively, based on the number of reverse protein sequence hits in the datasets. The relative quantitative data obtained were normalized and statistically analyzed using our in-house script as above (84).

### LC-MS/MS analysis

The setup of the μRPLC-MS system was as described previously (84). Chromatographic separation of peptides was carried out using an EASY nano-LC 1000 system (Thermo Fisher Scientific), equipped with a heated RP-HPLC column (75 μm × 37 cm) packed in-house with 1.9 μm C18 resin (ReproSil-AQ Pur, Dr. Maisch). Aliquots of 1-μg total peptides were analyzed per LC-MS/MS run using a linear gradient ranging from 95% solvent A (0.15% formic acid, 2% acetonitrile) and 5% solvent B (98% acetonitrile, 2% water, 0.15% formic acid) to 30% solvent B over 90 min at a flow rate of 200 nl/min. Mass spectrometry analysis was performed on a Q Exactive HF mass spectrometer equipped with a nanoelectrospray ion source (both Thermo Fisher Scientific). Each MS1 scan was followed by high-collision dissociation (HCD) of the 10 most abundant precursor ions with dynamic exclusion for 20 s. Total cycle time was approximately 1 s. For MS1, 3e6 ions were accumulated in the Orbitrap cell over a maximum time of 100 ms and scanned at a resolution of 120,000 FWHM (at 200 m/z). MS2 scans were acquired at a target setting of 1e5 ions, an accumulation time of 100 ms, and a resolution of 30,000 FWHM (at 200 m/z). Singly charged ions and ions with unassigned charge state were excluded from triggering MS2 events. The normalized collision energy was set to 35%, the mass isolation window was set to 1.1 m/z, and one microscan was acquired for each spectrum.

### Protein quantification and database searching

The acquired raw files were converted to the mascot generic file (mgf) format using the msconvert tool [part of ProteoWizard, version 3.0.4624 (2013-6-3)]. Using the MASCOT algorithm (Matrix Science, Version 2.4.1), the mgf files were searched against a decoy database containing normal and reverse sequences of the predicted SwissProt entries of Homo sapiens (www.ebi.ac.uk, release date 2014/11/24), the six calibration mix proteins (84) and commonly observed contaminants (in total 84,610 sequences for Homo sapiens) generated using the SequenceReverser tool from the MaxQuant software (Version 1.0.13.13). The precursor ion tolerance was set to 10 ppm, and fragment ion tolerance was set to 0.02 Da. The search criteria were set as follows: full tryptic specificity was required (cleavage after lysine or arginine residues unless followed by proline), three missed cleavages were allowed, and carbamidomethylation (C) and TMTsixplex (K and peptide n-terminus) were set as fixed modification and oxidation (M) as a variable modification. Next, the database search results were imported to the Scaffold Q+ software (version 4.3.2, Proteome Software Inc., Portland, OR) and the protein false identification rate was set to 1% based on the number of decoy hits. Protein probabilities were assigned by the Protein Prophet program (86). Proteins that contained similar peptides and could not be differentiated based on MS/MS analysis alone were grouped to satisfy the principles of parsimony. Proteins sharing significant peptide evidence were grouped into clusters. Acquired reporter ion intensities in the experiments were employed for automated quantification and statistical analysis using a modified version of our in-house developed SafeQuant R script, v2.3 (84). This analysis included adjustment of reporter ion intensities, global data normalization by equalizing the total reporter ion intensity across all channels, summation of reporter ion intensities per protein and channel, calculation of protein abundance ratios, and testing for differential abundance using empirical Bayes moderated t-statistics. Finally, the calculated p-values were corrected for multiple testing using the Benjamini−Hochberg method.

### ROS production assay of human neutrophils

ROS production was measured using luminol-enhanced chemiluminescence, as previously described (81). In brief, 2 × 10^5^ cells were incubated in RPMI+10% HS for 1 h at 37°C, 5% CO_2_ without inhibitors, or with 10 μM DPI. Neutrophils were stimulated with opsonized *Salmonella* (MOI=100 and 200) or *Candida albicans* (MOI=2) in the presence of 10% human serum and 100 μM luminol (Fluka) in HBSS (Invitrogen, Gibco) containing 0.1% glucose (Braun). Chemiluminescence was measured at 5-min intervals at 37°C with a luminometer (MicroLumat Plus, Berthold Technologies). Values were corrected based on unstimulated controls and initial time points.

### Phosflow assay with flow cytometry

p-Akt (pS473, BD Phosflow™, Cat. No. 558434), p-mTOR (pS2448, BD Phosflow™, Cat. No. 564242), p-p38 (pT180/pY182, BD Phosflow™, Cat. No 612595), p-STAT5 (pY694, BD Phosflow™, Cat. No 612598), and p-CREB (pS133, BD Phosflow™, Cat. No 558434) in human neutrophils were measured by flow cytometry using an adapted BD Phosflow™ protocol for human PBMCs. In brief, 500,000 PMNs were pretreated with inhibitors for 1 h, washed twice, and then stimulated with GM-CSF or PMA (Sigma, 10 nM, as positive control) for 30 min. The cells were fixed with 1× BD Phosflow™ Fix buffer I (BD, Cat. No. 557870) for 12 min at 37°C, and then permeabilized using BD Phosflow™ Perm buffer III (Cat. No. 558050) on ice for 30 min, followed by indicated Phosflow™ antibody staining for another 1 h at room temperature. Unspecific Fc receptor blocking (Human TruStain FcX™, BioLegend, Cat. No. 422301) and surface staining with FITC anti-human CD66b (Clone: G10F5, BioLegend) or APC anti-human CD66b (Clone: G10F5, BioLegend) for 1 h was performed immediately before fixation for 30 min. The stainings were initially performed with respective IgG isotype control (BD, Cat. No. 5577839). For data analysis, CD66^+^ cells were initially gated to check SSC/FSC position in FACS plot. Data were obtained using FACS CytoFLEX (Beckman Coulter) and analyzed using FlowJo v10.4.1.

### Surface molecule staining for flow cytometry

After PMN isolation or stimulation with cytokines, human neutrophils were washed twice in PBS to remove all cell debris and cytokines. To reduce unspecific antibody binding, neutrophils were incubated for 15 min at room temperature in the presence of human TruStain FcX™ Blocking solution (BioLegend, Catalog No.: 422302, 2 µl/test) followed by antibody staining for 30 min in the dark at 4°C. Samples were acquired with a BD Fortessa or CytoFLEX (Beckman Coulter) flow cytometer. Data were analyzed using FlowJo software, version v10.4.1. As a negative control, unstimulated sample or isotype control was used. Antibodies for PE anti-human HLA-DR/-DP/-DQ (clone: REA332, Miltenyi), PE-Cy7 anti-human HLA-DR (Clone: Tü39, BioLegend), APC anti-human HLA-DR (clone: L243, BioLegend), APC-Cy7 anti-human HLA-DR (clone: L243, BioLegend), FITC anti-human CD66b (clone: G10F5, BioLegend), APC anti-human CD66b (clone: G10F5, BioLegend), APC anti-human CD74 (clone: LN2, BioLegend), APC-Cy7 anti-human HLA-A, -B, -C (W6/32, BioLegend), FITC mouse isotype control IgM (clone: MM-30, BioLegend), APC-Cy7 mouse isotype control IgG1 (clone: MOPC-21, BioLegend), PE-Cy7 mouse isotype control IgG2a (clone: MOPC-21, BioLegend), APC mouse isotype control IgG1 (clone: MOPC-21, BioLegend), APC mouse isotype control IgG2a (clone: MOPC-21, BioLegend), APC-Cy7 mouse isotype control IgG2a (clone: MOPC-173, BioLegend), APC mouse isotype control IgG2a (clone: MOPC-173, BioLegend), and Zombie UV Fixable Viability Kit (BioLegend) were used.

### CD4^+^ T-cell clone activation *in vitro*

Antigen-specific CD4^+^ T-cell clones for the *A. fumigatus* 15-mer antigen Crf1/p41 were generated and expanded using the rapid expansion protocol as previously described (87, 88). Mature dendritic cells were generated from autologous PBMC as previously published (68, 89). Autologous PMNs were stimulated with GM-CSF (10 ng/ml) and IFN-γ (1 ng/ml) in the presence of Pan-Caspase OPH inhibitor Q-VD (3 µM) for 48 h. T-cell stimulation was analyzed by intracellular cytokine staining as previously published (40, 89). Briefly, DCs and PMNs were pulsed with 1 µg/ml Crf1/p41 peptide at 37°C for 30 min, and then washed twice to remove unspecific antigen. DCs and PMNs were co-incubated with the CD4^+^ T-cell clones (1 × 10^5^ cells) in a ratio of 1:1 and 10:1, respectively, in RPMI with 5% human serum supplemented with q.OPh Pan-Caspase inhibitor (3 µM) at 37°C for 6 h in the presence of brefeldin A (10 µg/ml) for the last 5 h. Cells were stained with Zombie Aqua Fixable Viability Dye (BioLegend, Cat. No. 423101), CD3-Brilliant Violet 785 (BioLegend, Cat. No. 317330), CD4-Brilliant Violet 650 (BioLegend, Cat. No. 317436), IFN-γ-APC (BioLegend Cat. No. 502512), and TNF-α-PE/Cy7 (BioLegend, Cat. No. 502930).

### Caspase activity assay

FAM-FLICA Caspase-3/-7 Assay Kit (Cat. No. 93), FAM-FLICA Caspase-8 Assay Kit (Cat. No. 99), FAM-FLICA Caspase-9 Assay Kit (Cat. No. 912), and FAM-FLICA Poly Caspase Assay Kit (Cat. No. 91) were ordered from ImmunoChemistry Technologies. Reagents were dissolved in DMSO as indicated by the manufacturer’s instruction. 1× FLICA Caspase solution was added to 300,000 neutrophils/condition in a volume of 100 µl for 30 min at 37°C, and then washed twice with FACS buffer and analyzed for its fluorescence in FL-1 channel with a flow cytometer (Accuri C6, BD).

### Caspase inhibition assay

Caspase inhibitors for Caspase-3, Caspase-6, Caspase-7, Caspase-8, and Caspase-9 were ordered from R&D (Sampler Pack, Cat. No. FMKSP01) and Pan-Caspase OPH inhibitor Q-VD (short q.OPh, R&D Systems, Cat. No. OPH001). In brief, 100,000 neutrophils/condition were pretreated with caspase inhibitors (final concentration = 10 µM for sampler pack inhibitors, final concentration = 3 µM for Pan-Caspase OPH inhibitor Q-VD) for 18 h, and then washed with FACS buffer and stained for PI (1:100) and Annexin V-APC (1:100) for 15 min at room temperature. Sample acquisition was done in between 2 h by using FL-2 (PI) and FL-4 (APC) on Accuri C6 flow cytometer (BD).

### Annexin V assay

Followed by antibody staining, the cells were washed and re-buffered in FACS buffer supplemented with 2.5 mM CaCl_2_, stained with Annexin V (1:100) for 15 min at room temperature and washed in FACS buffer. Sample acquisition was done in between 2 h. BV421 Annexin V (BioLegend), APC Annexin V (BioLegend), and FITC Annexin V (BioLegend) were used.

### Cytokine measurements in human plasma

Peripheral blood was drawn in 2.7-ml polyethylene tubes (Sarstedt) and spun down at 1,600 × g for 10 min, and plasma-rich supernatant (upper phase) was collected and stored at –80°C until further processing. Cytokines in plasma were measured using the customized human LEGENDplex™ multi-analyte flow assay kit (BioLegend) for IL-13, IL-2, GM-CSF, IL-9, IL-10, IFN-γ, TNF-α, IL-17A, IL-6, IL-4, IL-21, IL-2, and IL-17F, according to the manufacturer’s instructions.

### ImageStreamX

2 × 10^6^ cells were plated in a 96-well plate, followed by human TruStain FcX™ blocking for 15 min at 4°C (BioLegend, Catalog No.: 422302) and antibody staining (HLA-DR/DP/DQ-PE, Miltenyi Biotec, No. 130-104-827 and CD66b-AlexaFluor647, BioLegend, No. 305110) for 30 min in the dark at 4°C. Cells were fixed with fixation buffer (BioLegend, No. 420801) for 20 min at room temperature, permeabilized with permeabilization wash buffer (BioLegend, No. 421002), and stained with DAPI (final conc. 0.5 µg/ml) for 5 min at room temperature. Cells were resuspended in FACS buffer and acquired with ImageStreamX Mark II Imaging Flow cytometer (EMD Millipore) at 60× magnification using the channels CH1 (bright-field), CH3 (HLA-DR/DP/DQ-PE), CH7 (DAPI), and CH11 (CD66b-AlexaFluor647).

### mRNA determination and RT-PCR

Total RNA was extracted from 10 × 10^6^ human neutrophils at 4 h, 24 h, and 48 of incubation by using the RNeasy mini kit (Qiagen, Cat. No. 74104) according to the manufacturer’s instructions, with a second RNA purification step to optimize RNA yield and purity (90). The RNA concentration was measured with NanoDrop (Thermo Fisher Scientific), and purity was determined with the ratio 260 nm/280 nm method. cDNA synthesis was performed with the Omniscript RT kit (Qiagen, Cat. No. 2051119). mRNA expression was measured by real-time PCR using the FastStart Universal SYBR Green Master (Roche, Cat. No. 04 913 850 001). The deltadelta Ct method (91) was used to obtain the relative mRNA expression using GAPDH and B2M as internal controls (HK) (92). Samples were run in technical triplicates. Results are shown as fold change relative to unstimulated and internal control. The following primers were used: *CIITA* (forward: CTG AAG GAT GTG GAA GAC CTG GGA AAG C, reverse: GTC CCC GAT CTT GTT CTC ACT C), *B2M* (forward: ACT GAA TTC ACC CCC ACT GA, reverse: CCT CCA TGA TGC TGC TTA CA), *HLA-DRA* (forward: GAG TTT GAT GCT CCA AGC CCT CTC CCA, reverse: CAG AGG CCC CCT GCG TTC TGC TGC ATT), *CD74* (forward: CAC CTG CTC CAG AAT GCT G, reverse: CAG TTC CAG TGA CTC TTT CG), *RFX5* (forward: GTG TTT ATG ATG CCT ATC GGA AGT, reverse: TCC TCC TTA TGC CAC TGT AGC), *CREB* (forward: ATG GAA TCT GGA GCC GAG AA, reverse: GTG GCT GGG CTT GAA CTG), *RFXANK* (forward: TGA GAC CGT TCG CTT CCT, reverse: GTC CCT CCA TTC CAA TCA TAG ATG), *NFYa* (forward: GCC AGG CAA TGT GGT CAA, reverse: GCT TCT TCA TCG GCT TGG TT), *GAPDH* (forward: AAG TAT GAC AAC AGC CTC AAG AT, reverse: CAT GAG TCC TTC CAC GAT ACC), *NLRC5* (forward: GAG AGT GGA CCT GGA GAA GA, reverse: GCG GAT GAC TTG GAT GCT A), *HLA-B* (forward: TGA GAT GGG AGC CGT CTT, reverse: CAC GCA GCC TGA GAG TAG).

### Statistical analysis

Medians were used to present data from human samples. Comparisons between unpaired groups with a non-parametric distribution were made using the Mann–Whitney test. Comparisons between paired groups with a non-parametric distribution were made using Wilcoxon matched-pairs signed rank test. P values < 0.05 were considered statistically significant and indicated as follows: *P < 0.01; **P < 0.001; ***P < 0.0001; ****P < 0.0001. Statistical analysis was performed with GraphPad Prism 7 software.

## Data Availability

The raw data supporting the conclusions of this article will be made available by the authors, without undue reservation.
