# Supplementary material for: Unveiling signaling pathways inducing MHC class II expression in neutrophils
Source: Front Immunol. 2024 Sep 30;15:1444558. doi: 10.3389/fimmu.2024.1444558 (PMC11472776; doi:10.3389/fimmu.2024.1444558)
Supplement: Supplementary file 1 [file DataSheet1.pdf]

Supplementary Material

Supplementary Figures

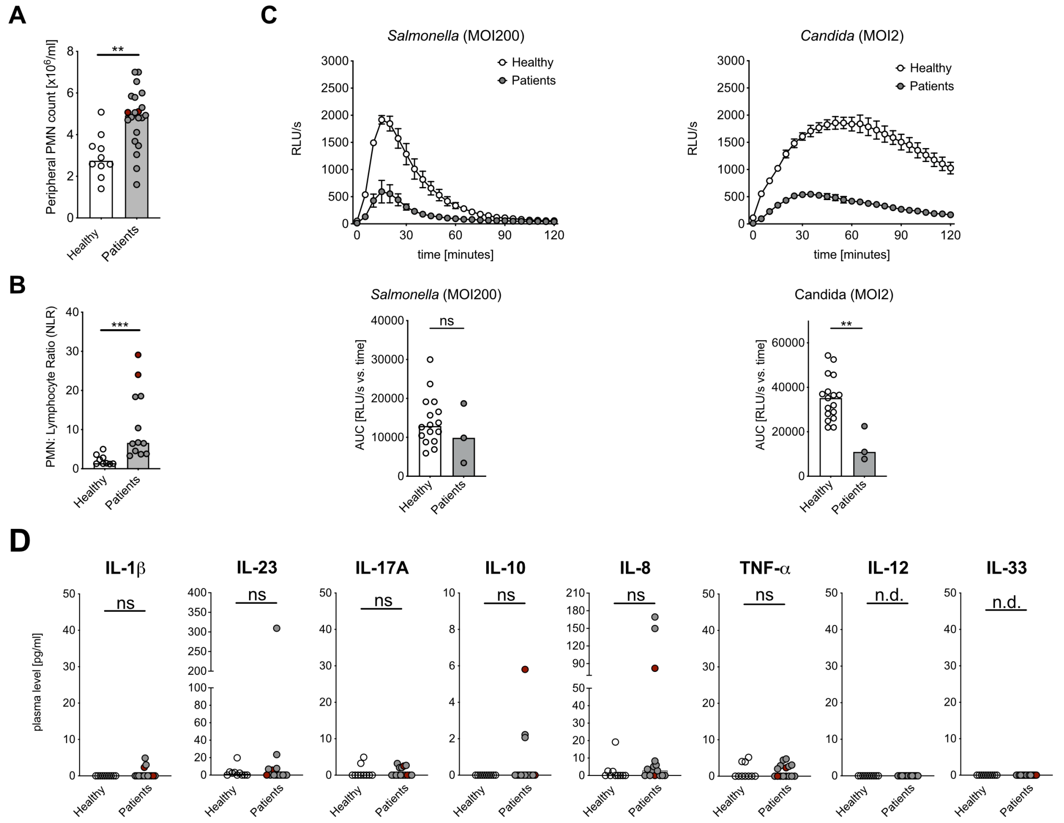

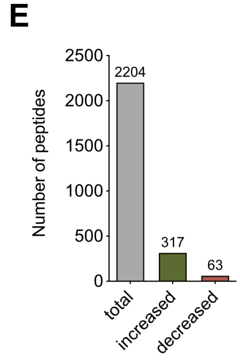

F

Antigen presentation by MHC class I and MHC class II

Uniprot ID	Protein	Fold Change (log2)	q-value
P14625	Endoplasmin (ENPL_HUMAN)	0.6	0.005
P07900	Heat Shock Protein HSP 90-alpha (HS90A_HUMAN)	0.61	0.008
Q92598	Heat shock protein 105 kDa (HS105_HUMAN)	2.19	0.002
P30480	HLA class I histocompatibility antigen, B-42 alpha chain (1B42_HUMAN)	0.61	0.013
Q31612	HLA class I histocompatibility antigen, B-73 alpha chain (1B73_HUMAN)	1.49	0.020
P30483	HLA class I histocompatibility antigen, B-45 alpha chain (1B45_HUMAN)	2.14	0.038
P27797	Calreticulin (CALR_HUMAN)	0.63	0.025
Q03518	Antigen peptide transporter 1 (TAP1_HUMAN)	1.52	0.007
Q15436	Protein Transport Protein Sec23A (SC23A_HUMAN)	0.66	0.017
Q9NZ08	Endoplasmatic reticulum aminopeptidase 1 (ERAP1_HUMAN)	1.10	0.037
O15533	Tapasin (TPSN_HUMAN)	0.88	0.017
P51159	Ras-related protein Rab-27A (RB27A_HUMAN)	0.63	0.002
Q9Y4L1	Hypoxia up-regulated protein 1 (HYOU1_HUMAN)	1.11	0.003
P12314	High affinity immunoglobulin gamma Fc receptor I (FCGR1_HUMAN)	1.67	0.028
P05771	Protein Kinase C beta type (KPCB_HUMAN)	0.74	0.039
P61163	Alpha-centractin (ACTZ_HUMAN)	0.6	0.029
Q15836	Vesicle-associated membrane protein 3 (VAMP3_HUMAN)	1.43	0.018
O94855	Protein transport protein Sec24D (SC24D_HUMAN)	1.28	0.004
O00232	26S Proteasome non-ATPase regulatory subunit 12 (PSD12_HUMAN)	1.19	0.013
P49721	Proteasome subunit beta type-2 (PSB2_HUMAN)	0.65	0.026
P28070	Proteasome subunit beta type-4 (PSB4_HUMAN)	0.67	0.044

Supplementary Figure 1. Neutrophils mimic APC-like phenotype in sepsis. **(A)** Human peripheral blood-derived neutrophil counts (PMN, in x10^6 cells) of healthy controls and patients. Medians, Mann-Whitney test. **(B)** PMN-to-lymphocyte ratio (NLR) as an index for severity of disease, measured in whole blood with flow cytometry using anti-CD66+ / -CD3+ antibodies. Medians, Mann-Whitney test. **(C)** ROS production (RLU/s detection over 2 hours) in response to *in vitro Salmonella* and *Candida* stimulation for healthy controls and bacteremia patients: representative kinetics (top), means and standard deviations; and statistical analysis (bottom), medians Mann-Whitney test). DPI (NADPH oxidase inhibitor, 10µM) was used as a control. ns, not significant. **(D)** Plasma cytokine concentration for IL-1β, IL-33, IL-23, IL-17A, IL-12, IL-10, IL-8 and TNF-α of healthy controls and patients. Medians, Mann-Whitney test; ns, not significant; n.d., not determined, out of standard curve range). **(E)** Number of peptides identified by human neutrophil proteomics from healthy controls and patients with significantly increased and decreased peptide levels. Significance threshold was set at *P-value* < 0.02. **(F)** List of significant protein changes involved in Antigen presentation by MHC class I and II from proteomics analysis. Significance threshold was set at *P-value* < 0.02 and fold change ≤ 1.5 cutoff in order of *P-value*. Target proteins were identified by using Metacore software analysis, Uniprot database (www.uniprot.org) and extensive literature research. MOI, multiplicity of infection; RLU, relative light unit; AUC, area under the curve; IL – interleukin; TNF, tumor necrosis factor.

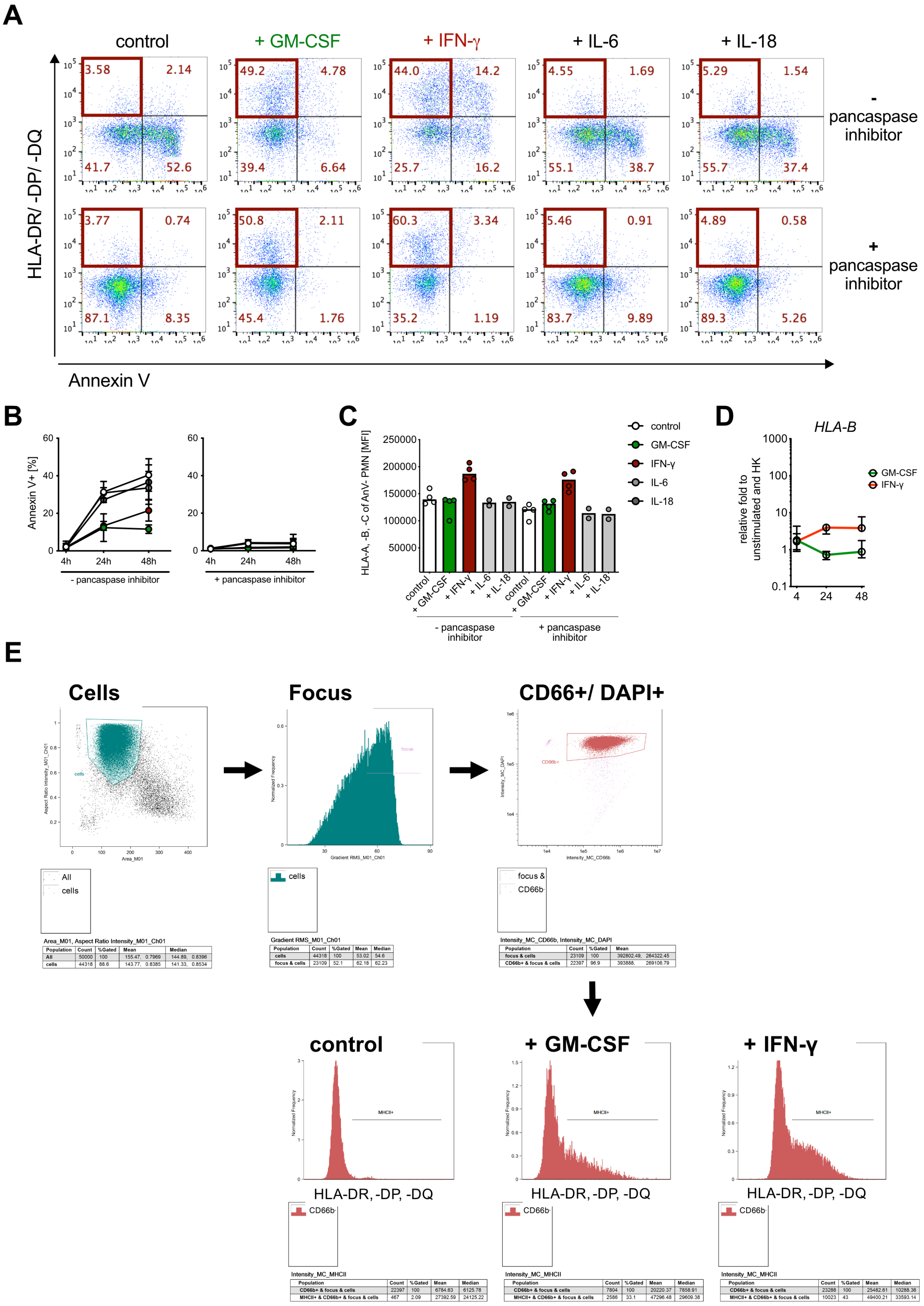
A
control
+ GM-CSF
+ IFN-γ
+ IL-6
+ IL-18
HLA-DR/ -DP/ -DQ
Annexin V
- pancaspase inhibitor
+ pancaspase inhibitor
3.58
2.14
41.7
52.6
49.2
4.78
39.4
6.64
44.0
14.2
25.7
16.2
4.55
1.69
55.1
38.7
5.29
1.54
55.7
37.4
3.77
0.74
87.1
8.35
50.8
2.11
45.4
1.76
60.3
3.34
35.2
1.19
5.46
0.91
83.7
9.89
4.89
0.58
89.3
5.26
B
Annexin V+ [%]
4h
24h
48h
- pancaspase inhibitor
+ pancaspase inhibitor
C
HLA-A, -B, -C of AnV- PMN [MFI]
control
GM-CSF
IFN-γ
IL-6
IL-18
+ GM-CSF
+ IFN-γ
+ IL-6
+ IL-18
- pancaspase inhibitor
+ pancaspase inhibitor
D
HLA-B
relative fold to unstimulated and HK
GM-CSF
IFN-γ
E
Cells
Focus
CD66+/ DAPI+
All
cells
focus &
CD66b
control
+ GM-CSF
+ IFN-γ
HLA-DR, -DP, -DQ
CD66b

Supplementary Figure 2. GM-CSF and IFN-γ induce APC-like phenotype in human neutrophils *in vitro* and reduce neutrophil apoptosis. (A) Representative scatter dot plots of HLA-DR/ -DP/ -DQ (MHC class II) and Annexin V surface expression on human neutrophils (CD66b^{+} cells) after stimulation with human recombinant GM-CSF (10 ng/ml), IFN-γ (10 ng/ml), IL-6 (10 ng/ml) and IL-18 (10 ng/ml) for 48h, pre-incubated -/ + Pan-Caspase inhibitor q.OPh (3µM) for 1h, measured with flow cytometry. **(B)** Apoptotic neutrophils (Annexin V^{+}) shown after stimulation with human recombinant GM-CSF (10 ng/ml), IFN-γ (10 ng/ml), IL-6 (10 ng/ml) and IL-18 (10 ng/ml) for 4h, 24h and 48h, pre-incubated with/ without Pan-Caspase inhibitor q.OPh (3µM), measured with flow cytometry. Medians (n=3-6). **(C)** HLA-A, -B, -C (MHC class I) expression (MFI) of viable (Annexin V^{-}) neutrophils was measured after stimulation with human recombinant GM-CSF (10 ng/ml), IFN-γ (10 ng/ml), IL-6 (10 ng/ml) and IL-18 (10 ng/ml) for 24h, pre-incubated with/ without Pan-Caspase inhibitor q.OPh (3µM), measured with flow cytometry. All values are shown as medians (n=2-4). **(D)** *HLA-B* (one of the MHC class I alleles) mRNA expression after stimulation with human recombinant GM-CSF (10 ng/ml) and IFN-γ (10 ng/ml) for 4, 24 and 48h. Values are shown as relative fold change to unstimulated control and internal control (housekeeping gene, HK). All values are shown as medians (n=6) **(E)** Gating strategy of a representative example of *de novo* MHC class II (HLA-DR/ -DP/ -DQ) surface molecule expression on neutrophils after stimulation with human recombinant GM-CSF (10 ng/ml) respectively IFN-γ (10 ng/ml) for 48h, measured by Image Stream X. The cell population gate was defined by the Aspect Ratio Intensity and Area, the focus was set at Gradient RMS levels > 50. Human neutrophils were identified as CD66+/DAPI+ cells.

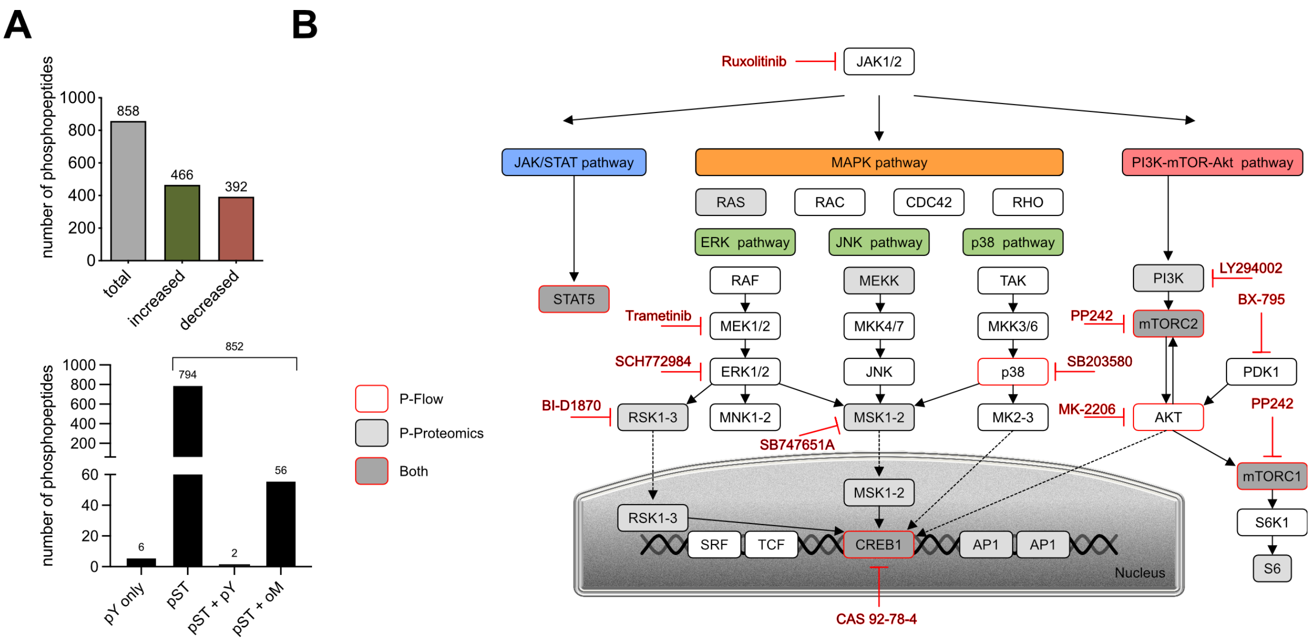

Supplementary Figure 3. GM-CSF signaling leads to the activation of JAK-STAT, MAPK p38 and mTOR-AKT signaling pathways and phosphorylation of transcription factor CREB. (A) Top: significantly changed phosphopeptides detected in neutrophils of healthy donors after stimulation with GM-CSF for 30 min (from total 1328 phosphoproteins and 3579 phosphhopeptides). Phosphoproteomics data were performed in three biological replica each condition. Significance

threshold was set at *q-value* < 0.05 and fold change ≤ 2 cutoff in order of *P-value*. **Bottom:** Significantly changed phospopeptides by the type of phosphorylation. **(B)** Overview of hierarchical signaling phosphorylation events (highlighted according to the legend on the left) in human neutrophils upon GM-CSF stimulation *in vitro*. Phosphorylation of proteins confirmed by PhosFlow only are highlighted by a red frame, by phosphoproteome only – filled in grey, confirmed by both methods – by red frame and dark-grey filling. JAK1/2 (Janus Kinase 1 and 2) inhibition by Ruxolitinib inhibits all three different pathways. The inhibitors tested are shown in black and red font next to respective proteins. STAT5, Signal Transducer and Activator of Transcription 5; p38, mitogen-activated protein kinase; MSK1, Mitogen- and stress-activated protein kinase; PI3K, Phosphoinositidine-3-kinase; mTORC1 and mTORC2, mammalian target of rapamycin complex 1 and 2; S6K1, p70 ribosomal S6 kinase; RSK1-3, p90 ribosomal S6 kinase 1 and 3; JNK, C-Jun-N-terminal kinase; RAS, Rat sarcoma protein; RAC, subfamily of Rho GTPases (guanosine triphosphatases); CDC42, Cell division control protein 42; TAK, Tat-associated kinase; MEKK, MAP kinase kinase kinase; MEK, kinase of MAP kinase; MKK, Mitogen-activated protein kinase kinase; ERK, Extracellular signal-regulated kinase; MK, Mitogen-activated protein kinase 2; PDK1, Pyruvate Dehydrogenase Kinase 1; MNK1-2, MAPK interacting protein kinases 1 and 2, MK2-3, MAPK-activated protein kinases 2 and 3; SRF, Serum response factor (a transcription factor), TCF, T cell factor (a transcription factor); AP1, Activator protein-1 (a transcription factor); CREB1, cAMP responsive element binding protein 1 (a transcription factor).

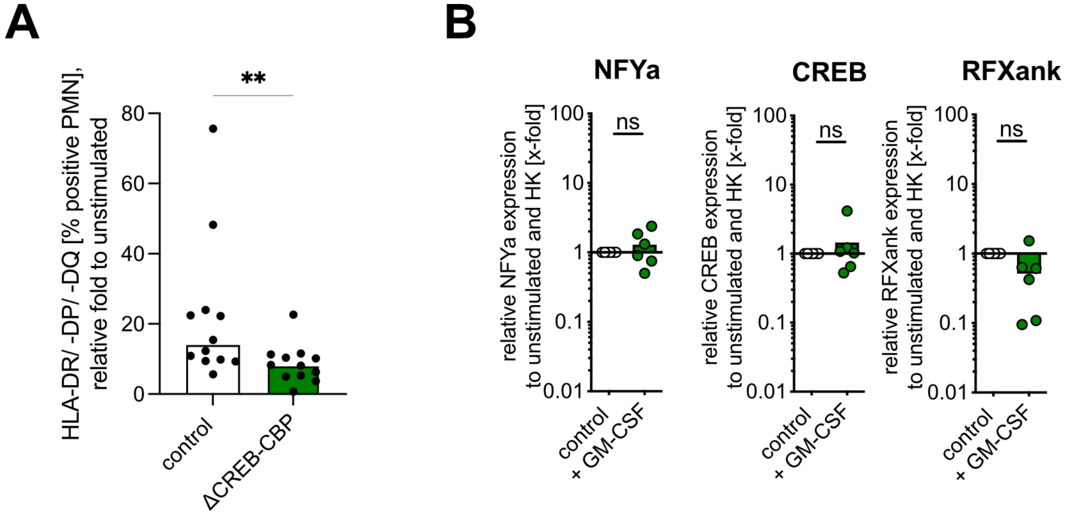

Supplementary Figure 4. Targeting the MHC class II enhanceosome in human neutrophils

(A) HLA-DR/ -DP/ -DQ^{+} (MHC class II^{+}) /Annexin V^{-} neutrophils after pre-incubation with/ without CAS 92-78-4 inhibitor (CAS 92-78-4 (ΔCREB-CBP interaction inhibitor), final concentration 100µM) and stimulation with human recombinant GM-CSF (10 ng/ml) for 48h. Medians, Wilcoxon signed rank test. **(B)** *NFYa, CREB* and *RFXank* mRNA expression after stimulation with human recombinant GM-CSF (10 ng/ml) for 4h. Values are shown as relative fold change to unstimulated control and internal control (housekeeping genes, HK). Medians, Wilcoxon signed rank test; ns, not significant. CREB, cAMP responsive element binding protein; CBP, CREB-binding protein; NFYa, Nuclear transcription factor Y subunit alpha, RFXank, Regulatory Factor X Associated Ankyrin Containing Protein.
